# Ligation of Primitive Carotid Artery for Traumatic Aneurism

**Published:** 1884-01-20

**Authors:** Jno. Thad. Johnson

**Affiliations:** Professor of Surgery, Southern Medical College, Atlanta, Georgia


### Southern Medical Record:

EDITORS:

THOMAS S. POWELL, M.D.   R. C. WORD, M.D.

*R. C. WORD, M.D., Managing Editor.*

☞All Communications and Letters on Business connected with the RECORD must be addressed to the Managing Editor.

VOL. XIV.   ATLANTA, GA., JANUARY 20, 1884.   No. 1.

### ORIGINAL AND SELECTED ARTICLES.

### LIGATION OF PRIMITIVE CAROTID ARTERY FOR TRAUMATIC ANEURISM.

BY JNO. THAD. JOHNSON, M. D

Professor of Surgery, Southern Medical College, Atlanta, Georgia

Mr. C., on December 1st, in endeavoring to aid a friend who had become involved in a dispute with a negro, received a penetrating wound from the blade of "an ordinary pocket-knife," on the side of the neck, about an inch below the angle of the jaw. The incision was one inch and a quarter in length, and one in depth. The hemorrhage was free, amounting, probably, to a quart, and was arrested by pressure of the fingers of his friend, directly on the wound. It had entirely ceased when the physician, Dr. T. D. Longino, arrived. Four hours later, however, after the physician had departed, the bleeding recommenced, and. after the loss of about one pint, was again controlled by pressure, this time at the hands of Dr. T. E. Pennington who, happily, was now present.

The wound, Dr. Longino, the physician attending, informs me, united by first intention, except the extent of one-eighth of an inch. The swelling now almost disappeared, though not entirely.

The Doctor felt anxious all the while as to possible results ; but not until the eighth day was any abnormality really discovered.

The tumor was now not larger than a filbert, but grew steadily from this time on to the 20th of December.

On this latter date a hemorrhage occurred, a half-pint in amount, and was controlled by his wife, by pressure applied to the portion of the wound that had failed of union—this, of course, being the first point to give way in the steady progress of the tumor's growth and pressure.

From the 21st to the 23d, the tumor almost doubled in size. The surface was so congested, even to the color of a purple, the covering seemed so thin, the ulcerated spot seemed so open, the pulsation so marked, the *whir* so pronounced and so *near*, that there was good ground for the fear that any heart-throb might burst the thin barrier interposed between his life and eternity.

The patient was kept in bed some days after receiving the stab. On the 19th he walked a half-mile; had done the same thing two or three days previous to this. After about the 10th of the month, though, his face bore a serious and anxious expression that might be expected as a consequence of a suspicion on his part, that all was not well, and that some calamity might befal him at any moment.

On the night before the operation (to be described) there was considerable oozing of blood, probably a couple of ounces, as reported by those who remained in the room with him through the night.

On the afternoon of December 23d I was summoned by telegram to see the case with Drs. Longino and Mixon. We were all soon agreed that the swelling was an aneurism of one of the three carotids; the swelling being directly over the point of bifurcation of the left primitive. The whir or murmur, was distinct. Pressure of the vessel lower down, against the transverse processes of the vertebræ produced perceptible diminution in size.

We were unanimous as to the necessity of promptly ligating. How could we be otherwise, with the danger so emphasized by repeated bleedings! The case, in all its bearings, was set before the patient and his friends, for their decision, and resulted promptly in a submission to our judgment.

It was now night, and as we would remain in the house during the night, and so could afford ready assistance in case of emergency, it was deemed best to await the morning, both for the benefit of its light and for avoiding the danger of firing the ether vapor.

During the night the family sent to Fayetteville, twelve miles distant, for Dr. Paul Faver, a friend and the former physician of the family. He arrived in the morning, examined the patient, and fully concurred in our decision.

Mr. C., the patient, was 35 years of age, six feet high, weighed

225 pounds and therefore was of heavy build—what has been termed the apoplectic form.

After due preparations were made the patient was placed on a firm table, and Dr. Longino commenced the administration of ether (Squibb's). After a few moments the patient began to evince the usual restlessness, then became nauseated (still conscious), and was turned upon his right side to facilitate vomiting. At the first straining, (not very violent) the aneurismal sac burst. A stream of blood seemingly as large as a man's thumb, was sent flying to the distance of five or six feet. So great, indeed, was the gush that for a fraction of a second all thought it was a liquid ejected from the stomach. In an instant Dr. Faver had thrust his fingers down in the sac, I had made compression of the vessel against the vertebræ below the aneurism, and the bleeding was completely arrested.

That we might proceed with the etherizing, pledgets of linen were pushed firmly in the sac, so as to prevent further bleeding. The ether did not act kindly ; on the contrary, he several times ceased to breathe, and considerable effort was required for his reviving. At such times the tongue had a tendency to fall back, (though not to the extent as with chloroform), the jaw became fixed, the face became livid, and the pulse was not discoverable. These symptoms constantly recurring, afforded the greatest anxiety throughout, and demanded Dr. Longino's utmost care and watchfulness.

In addition to the bleeding, and the threatened danger from the ether, his neck was thick, short, and fat, requiring a deep and difficult cut to reach the vessel. No great trouble, however, was encountered, beyond the expenditure of something more than the usual care ; the operation, of itself, required but a few minutes, but several stoppages were enforced from the causes above hinted at. The vessel was encircled just above the omo-hyoid muscle. The nerves were drawn carefully aside, hence suffered no injury.

We were now about to rejoice that the ether could be withheld, and the wound closed. But other difficulty was to be encountered. Withdrawing the lint from the sac, the blood was found to flow freely from the distal extremity of the divided vessel. It was not to be compared in quantity to the bleeding of a few minutes previous ; nor was there any jetting or spurting ; but yet it welled up so freely as to persuade us that the man's life would be a short one were the vessel left to itself for checking.

The little opportunity to search for the bleeding point ; the doubt as to whether the flow came from a point above or below the bifurcation, and if the latter, whether from the external or internal branch ; the impracticability of effective compression (except as with the fingers in the wound, which of itself entirely prevented any further operative step) ; the alarming nature of his present condition ; all this made such search, to say the best of it, but an unpromising one. We quickly decided to pack the cavity with lint saturated with tincture of iron (the only styptic at hand, aud one regarded as valuable as any). The cavity was so filled, the lint then drawn out and the process repeated two or three times, and until our few fluid ounces of the styptic were exhausted. The most of this was executed by Dr. Faver. Several silver wire sutures were firmly and carefully placed, closing the flaps neatly over the packing and these further supported by plaster strips.

The patient was placed in bed in a state of considerable shock, though it could scarcely be called an alarming one. Attempt was made to administer slight stimulants by the mouth, though with but indifferent success. He vomited, or made ineffectual efforts, several times within a short while after reaching the bed. One-eighth grain of morphia was given hypodermically, and acted well both as a stimulant, and in quieting the struggles of the stomach ; and he now was in even better condition than one would suppose.

I was compelled to leave very soon in order to reach my home-bound train. Dr. Faver left a little later. Drs. Longino and Mixon kindly consented to remain in charge ; Dr. Mixon was with him the greater part, and Dr. Longino all of the time until the end.

Several hours after the operation, probably during the night, the patient was discovered to be hemiplegic. It is possible such was his condition from that time. We did not examine for paralysis before my rather hurried departure.

The breathing increased gradually, as I learned from the physicians attending, from normal until it reached forty-two per minute, twenty-four hours after the operation. Morphia hypodermically reduced this to thirty-three; followed, however, after three hours, by an increase to forty-eight, at which it remained for several hours preceding his death. The temperature was not recorded.

His pulse, since the day of the wound, as observed by Dr. Longino, was remarkably intermittent and irregular. His brother informed Dr. L. that this had always been its character. After the operation it increased in frequeney and in feebleness, and possibly in its intermissions. The pulse-beat was at the rate of from 120 to 140, from the time of the operation until death. I do not know the cause of the intermittent pulse ; if from heart disease, this might account for the unfavorable symptoms provoked by the ether. He was probably conscious throughout, but gave no evidence of it, owing to the paralysis.

His death occurred thirty-six hours after the operation, he having been comatose for the last six or eight of these. I was more than usually anxious to obtain the privilege of an autopsy, but unfortunately it was denied us.

How unfortunate. let me digress to remark, that the thoughtless prejudice of friends, from a mistaken view of the respect due the deceased, should deprive science of information that might be so readily obtained, and yet. at times, of such inestimable value! No further disfiguring would have resulted in this case; as indeed, there usually is no necessity for unsightly cuts. Were we in the habit of making such examinations more frequently, the laity would become accustomed to the so-called *cutting up* of a corpse, and instead of a flat and hasty refusal, would take time to consider more kindly the necessity therefor; and then such a request, properly preferred, with the reason for our desire clearly, gently, briefly explained, would less often be met with refusal.

I cannot refrain from publicly thanking, as I heartily did more privately, the physicians who assisted me. An operator could not desire more zealous and efficient assistants than I found in the persons of each of the three, Drs. Faver, Longino, and F. T. Mixon.

*Atlanta, January, 1884.*

